# Relevance of the type III error in epidemiological maps

**DOI:** 10.1186/1476-072X-11-34

**Published:** 2012-08-18

**Authors:** Harald Heinzl, Thomas Waldhoer

**Affiliations:** 1 Center for Medical Statistics, Informatics, and Intelligent Systems, Medical University of Vienna, Vienna, Austria; 2 Department of Epidemiology, Center for Public Health, Medical University of Vienna, Borschkegasse 8a, A-1090, Vienna, Austria

**Keywords:** Directional test decision, Statistical power, Infant mortality, Standardised mortality ratio (SMR), Crude SMR estimator, Unstructured random effect, Structured random effect, BYM model

## Abstract

**Background:**

A type III error arises from a two-sided test, when one side is erroneously favoured although the true effect actually resides on the other side. The relevance of this grave error in decision-making is studied for epidemiological maps.

**Results:**

Theoretical considerations confirm that a type III error may be large for regions with small numbers of expected cases even when no spatial smoothing has been performed. A simulation study based on infant mortality data in Austria reveals that spatial smoothing may additionally increase the risk of type III errors.

**Conclusions:**

The occurrence of a type III error should be taken into account when interpreting results presented in epidemiological maps, particularly with regard to sparsely populated regions and spatial smoothing.

## Background

**Figure 1:**
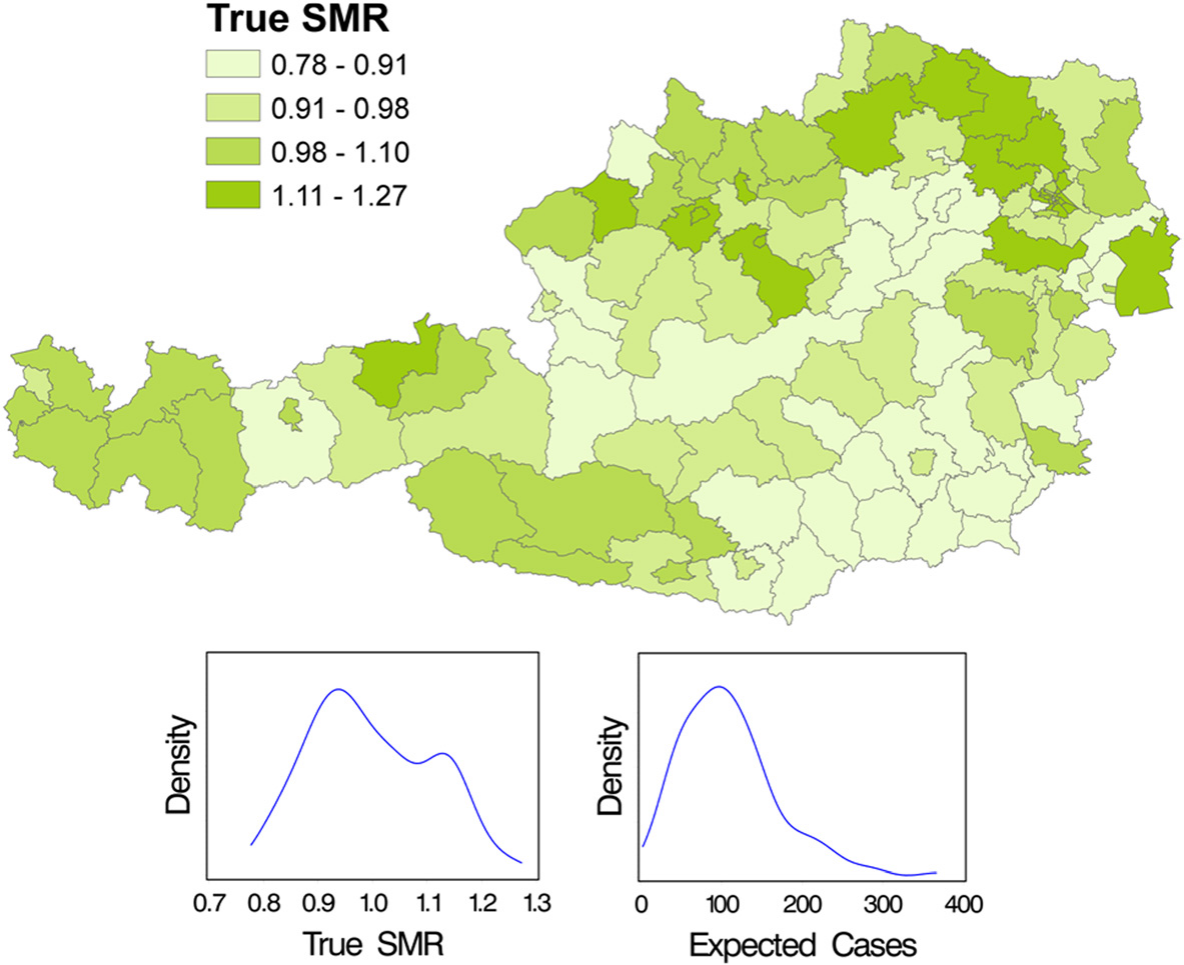
**Austrian infant mortality from 1984 to 2008 at the level of 121 administrative districts.** SMRs have been estimated by the empirical Bayes procedure and have been grouped into quartiles. The expression "true SMR" refers to the use of these results as calibration reference points for type III error calculations in the example. Kernel estimates of the distributions of SMRs and expected cases are also shown.

Epidemiological maps, also known as spatial maps or choropleth maps, are widely used, especially since the advent of powerful and user-friendly geographic information system (GIS) software tools. Among other aspects, public health indicators and health care performance measures are shown in graphic form on the basis of these maps. By way of an example, Figure 1 shows standardised mortality ratios (SMRs) of infant mortality across 121 Austrian districts.

SMR is a common epidemiological indicator for presenting and studying mortality in a spatial context. Three approaches to estimate the SMR will be considered in the following: crude, unstructured, and Besag-York-Mollié (BYM) [1].

The *crude* SMR is obtained by simply dividing the number of observed cases of a spatial unit by its corresponding number of expected cases. For the purpose of generalisability, it would be meaningful to consider crude SMR as being based on a simple Poisson model. Thus, the *unstructured* SMR may be considered to be based on a Poisson model, including a spatially unstructured random effect. BYM SMR is based on a Poisson model, including a spatially unstructured and a spatially structured random effect [1]. However, a Poisson model with a spatially structured random effect alone (i.e. a structured SMR approach), such as the conditionally autoregressive (CAR) model, is not considered in the present report.

In the simplest form the expected cases are derived by multiplying the overall nation-wide mortality rate with the number of population years of the spatial unit of interest. More refined approaches utilise the available covariate information as well.

The variability of the crude SMR estimator strongly depends on the size of the population of the respective spatial unit. This may yield extreme estimates, especially for sparsely populated spatial units. Nowadays the crude SMR is rarely used in spatial epidemiology. However, as it is the origin of all types of SMR estimators, it will be studied here for the purpose of comparison.

The incorporation of spatially unstructured and/or spatially structured random effects into SMR estimation is also known as spatial smoothing. The concept underlying spatial smoothing is "borrowing strength" from neighbouring spatial units in order to avoid extreme SMR estimates by flattening out random noise fluctuations. In practice, the computational implementation of spatial smoothing is usually performed in the context of a Bayesian statistical approach.

The question now arises as to whether and to what extent bias is introduced by spatial smoothing. A specific and particularly severe form of bias would be effect reversing: a spatial unit with a truly hazardous health effect for its inhabitants would yield an advantageous result in the epidemiological map, and vice versa. A statistically significant effect reversing is known as an error of the third kind or a type III error in statistical terms [2]. If a true health effect is present, the so-called *q*-value will be the conditional probability for a type III error, provided a statistically significant result has been obtained [3].

The present report addresses the practical relevance of a type III error in unsmoothed and spatially smoothed epidemiological maps by theoretical considerations and a simulation study, which is based on the actual data concerning infant mortality in Austria. The paper is organised as follows: Numerical and simulation results of a type III error are presented in the results section. These results and further aspects of the issue are discussed in the discussion section. Major findings are summarised and presented in the overall context in the conclusions section. The methods section addresses the infant mortality situation in Austria, the employed epidemiological models, the type III error, and the simulation study.

Note that the term *type III error* has another meaning as well. It is sometimes used to describe a mishap during statistical consulting when *the right answer is given to the wrong problem*[4]. Throughout the present report, however, the term *type III error* will always refer to *statistically significant effect reversing*.

## Results

The type III error will first be exemplified for the crude SMR estimator. The problem will then be studied in the context of epidemiological maps by applying different SMR estimators to Austrian infant mortality data in the framework of a simulation study.

### Exact results for crude SMR estimator

Assuming a Poisson distribution for the number of observed cases permits exact calculation of directional power, type III error, and the *q*-value for the crude SMR estimator.

The null hypothesis to be tested is whether the true SMR equals a value of one. The level of significance was set to α=0.05.

**Figure 2:**
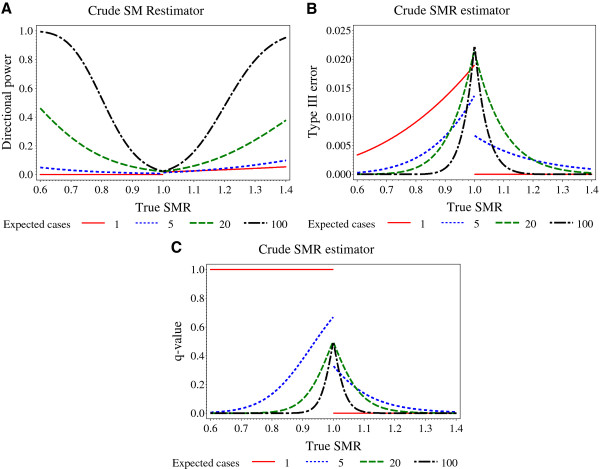
**Directional power (2a), type III error (2b) and*q*-value (2c) of the crude SMR estimator are plotted against the true SMR value for various numbers of expected cases.** The level of significance was set to α=0.05 .

In Figure 2a the directional power is plotted against the true SMR value for various numbers of expected cases. As anticipated, the directional power depends on the true SMR as well as the number of expected cases. The closer the true SMR is to a value of one, the smaller is the directional power. Ideally, the directional power should converge to half the significance level here. However, due to the discreteness of the exact Poisson test, directional power values considerably smaller than α/2=0.025 are observed especially for small numbers of expected cases. Larger numbers of expected cases are commonly associated with larger power values. Exceptions may occur for true SMR values close to one. These exceptions are again due to the discreteness of the exact test. Notably, if the number of expected cases is one the directional power is zero for true SMR values smaller than one. In other words, if the number of expected cases is one, the exact Poisson test can never result in a statistically significant crude SMR estimate smaller than one.

Figure 2b shows type III errors. For large numbers of expected cases the type III error is small. It is practically non-existent for true SMR values not too close to one. Discreteness effects are also observed.

Figure 2c shows *q*-values, i.e. the probability of a type III error when a statistically significant result is obtained. The *q*-value of one for a number of expected cases of one and true SMR values smaller than one is most impressive. In other words, whenever a statistically significant result is obtained for this scenario, it is always due to effect reversing.

### Results for Austrian infant mortality data

For calculation of statistical power, type III error and the *q*-value, the true underlying SMR must either be known or a reasonable value must be assumed. The latter particularly applies to sample size calculation [3].

In the following, infant mortality data across 121 Austrian districts are used to exemplify the type III error. Naturally, the true spatial distribution of Austrian infant mortality rates is unknown. Therefore, substitutes for true SMR values are used; these are empirical Bayes estimates based on infant mortality data from 1984 to 2008 (Figure 1).

#### Crude SMR estimator results for infant mortality data

**Figure 3:**
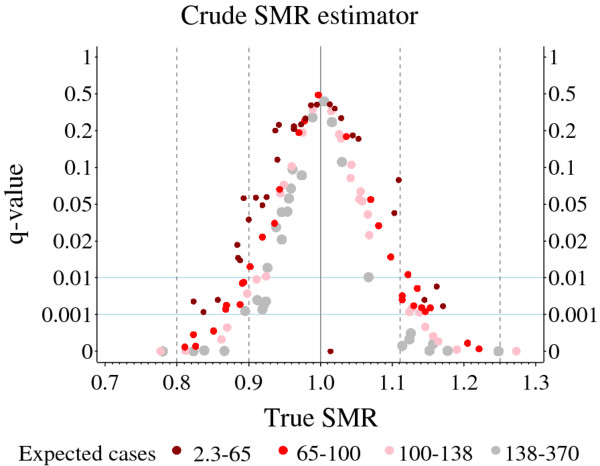
**Austrian infant mortality example: The*q*-values of the crude SMR estimator.** The level of significance was set to α=0.05 .

Analogous to the results presented in Figure 2a-2c, directional power, type III error and the *q*-value of the crude SMR estimator can be exactly calculated for Austrian infant mortality as well. Figure 3 shows a plot of the resulting *q*-values against the (assumed) true SMR values. The level of significance was set to α=0.05.

For the sake of clarity, the ordinate has been stretched for the lower values and squeezed for the larger ones. Horizontal light blue lines indicate selected *q*-values of one per mil and one per cent. The neutral true SMR value of one is shown as a vertical grey solid line. Vertical grey dashed lines at 0.8, 0.9, 1.111, and 1.25 are used to mark true SMR values with minor, average, and considerable deviation from one, respectively.

Despite different scalings of the ordinates, it is evident that the results in Figure 3 are in accordance with those in Figure 2c. The further afar the true SMR value is from one, the smaller is the *q*-value. Smaller *q*-values may also be expected for larger numbers of expected cases.

The dark red dot for a true SMR around 1.01 and a *q*-value of zero refers to a small district with a number of expected cases of around 2.3 (Figure 3). Here, a type III error cannot occur for the crude SMR estimator when the significance level is set to five per cent.

#### Results of random effect models for infant mortality data

Directional power, type III error and the *q*-value cannot be analytically calculated for SMR estimates of random effect models in general. SMR estimates for the i-th unit depend on the SMR estimates of its neighbouring units. As the neighbours themselves have neighbours, the random effect estimates are mutually interwoven. Therefore, directional power, type III error and the *q*-value of random effect models have been computed by computer simulations using the statistical software package R [5] in the context of a Bayesian statistical approach.

In order to compute "significantly" increased or decreased areas from the posterior distribution, common reference thresholds $\Delta_{01}=\Delta_{02}=1$ for the SMRs were used [6]. The cut-off probabilities $\omega_{\;1}$ and $\omega_{\;2}$ were set to values of either 0.8 or 0.975, i.e. either $\omega_{\;1}=\omega_{\;2}=0.8$ or $\omega_{\;1}=\omega_{\;2}=0.975$.

**Figure 4:**
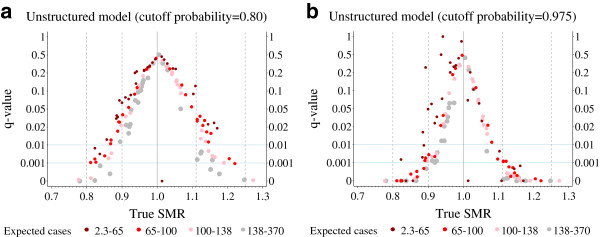
**Austrian infant mortality example: The*q*-values of the unstructured model.** Two different Bayes decision rules based on different cut-off probabilities [80% (**4a**) and 97.5% (**4b**)] are used.

For the unstructured model and a cut-off probability of 0.8 the *q*-values are plotted against the (assumed) true SMR values in Figure 4a. Apart from a general inclination in favour of higher *q*-values, there is hardly any appreciable difference compared to the results for the crude SMR estimator (Figure 3). Increasing the cut-off probability to 0.975 decreases *q*-values in the main (Figure 4b). Some districts with small numbers of expected cases constitute exceptions. This is due to the definition of the *q*-value, which relates "significant" effect reversing to all "significant" results. When the cut-off probability is increased, the proportion of "significant" results in any direction will generally decrease and the proportion of "significantly" effect-reversed results is expected to decrease even further. The latter is not always the case for some districts with small numbers of expected cases.

**Figure 5:**
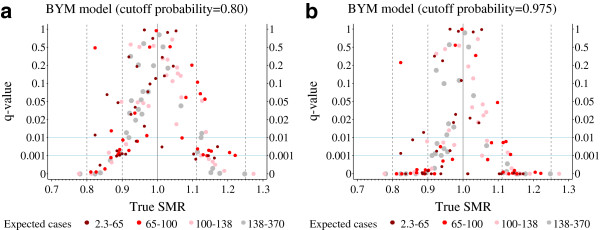
**Austrian infant mortality example: The*q*-values of the BYM model.** Two different Bayes decision rules based on different cut-off probabilities [80% (**5a**) and 97.5% (**5b**)] are used.

Figure 5a and 5b show *q*-values of the BYM model. Local smoothing obviously has an effect on the type III error. Interestingly, this effect is concentrated in districts with true SMR values that only marginally deviate from one.

**Figure 6:**
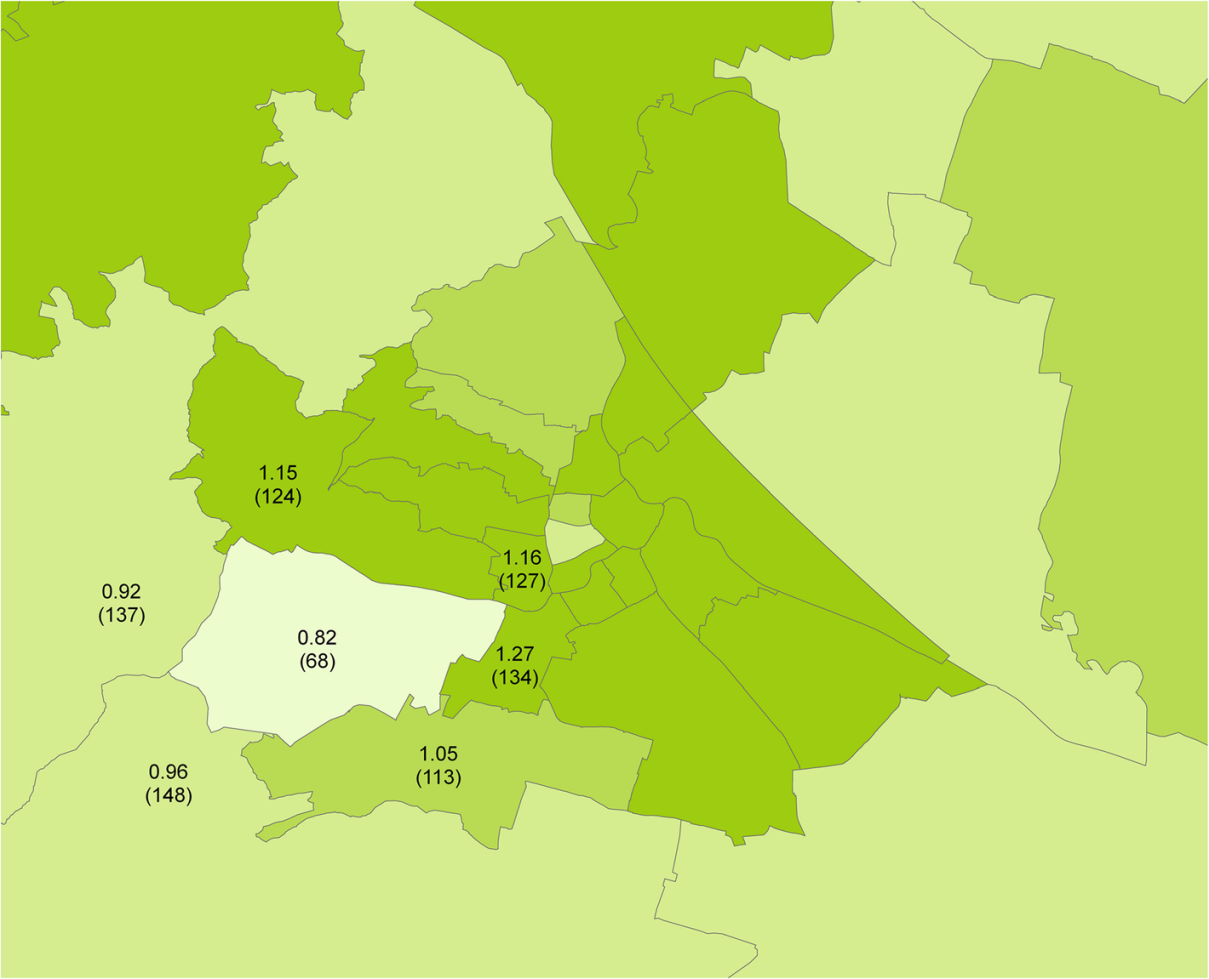
**Magnified segment of Figure**1**, showing empirical Bayes-estimated infant mortality SMRs in the Austrian capital of Vienna and its neighbouring districts.** The Viennese district with a markedly low infant mortality in the lower left quadrant is Hietzing. The SMR and the number of expected cases (in parentheses below) were numerically recorded for Hietzing and its neighbours.

A district with a rather small true SMR value of around 0.82 and *q*-values of 49% and 24%, as shown in Figure 5a and 5b, is worthy of mention. It is the Viennese district of Hietzing with 68 expected cases (Figure 6). The small true SMR value for Hietzing is an exception when compared to the SMR values of the surrounding districts. Obviously, the local shrinkage component of the BYM model is now responsible for considerable effect reversing. Without local shrinkage, effect reversing is of no relevance for Hietzing (Figures 3, 4a, 4b).

The *q*-value is calculated from the type III error and non-directional power. It is interesting to consider these values for the BYM results of Hietzing. For a cut-off probability of 0.8 (Figure 5a), the type III error and the non-directional power are 13% and 26%, respectively. These values decrease markedly when the cut-off probability increases to 0.975 (Figure 5b). Now the type III error and the non-directional power are 0.18% and 0.73%, respectively.

## Discussion

A type III error is due to random fluctuations in the first place. Translating structural assumptions in regard of the spatial dependency of data into a statistical model can create bias which, among other aspects, will increase the type III error problem. This has been demonstrated for epidemiological maps by comparing a spatially unstructured model (Figure 4a and 4b) with the BYM model (Figure 5a and 5b), where a spatially structured component was added to the latter.

For a given model, the type III error depends on the unknown true SMR as well as the number of expected cases (Figures 2, 3, 4, 5).

A true SMR close to one is generally associated with a rather large type III error, but with minor relevance. The further away the true SMR is from one, the more relevant though less likely will be the occurrence of a type III error. The dividing line between relevance and minor relevance is hard to define, and the appraisal of a relevant SMR deviation from one may vary from case to case. Note that a similar issue arises in the field of equivalence testing, where an equivalence range of (0.8, 1.25) was suggested for SMRs with reference to a traditional choice in bioequivalence trials [7].

In general, the type III error will become smaller as the number of expected cases increase. However, the relationship between expected cases and a type III error can become complex for the exact test of the crude SMR. For true SMR values larger than one, there may be no type III error for small numbers of expected cases due to the sheer numerical impossibility of obtaining a statistically significant result, and the functional dependence of a type III error on the expected cases can be sawtooth-shaped, as is frequently observed for power functions of exact tests in general (e.g. see Figure 4 in [3]).

The spatially smoothed results of districts with small numbers of expected cases are naturally susceptible to domination by neighbours with larger numbers of expected cases. This may even lead to reversal of the sign and, subsequently, to a bias-induced increase in type III error for the respective district.

Although a type III error is usually small in size, it should always be related to the probability of a significant result, i.e. the non-directional power [3]. This so-called *q*-value quantifies the risk for a type III error when a significant result has been obtained. This risk may be unacceptably high, especially for small numbers of expected cases.

It is obvious that both the reference thresholds $\Delta_{01}$ and $\Delta_{02}$, und the cut-off probabilities $\omega_{\;1}$ and $\omega_{\;2}$, may considerably influence the results of the Bayes approaches [6]. For the sake of simplicity $\Delta_{01}$ and $\Delta_{02}$ were set to one and $\omega_{\;1}$ and $\omega_{\;2}$ were varied together. As expected, a more liberal decision rule with $\omega_{\;1}=\omega_{\;2}=0.8$ leads to larger type III errors and *q*-values than a more conservative one with $\omega_{\;1}=\omega_{\;2}=0.975$.

Bayesian approaches could be rendered even more flexible by explicitly defining a loss function to address the various consequences of the various types of error. This does not only signify explicit weighting of the trade-off between type I, type II, and type III error; it could also include weighting of a type III error depending on its direction, because obtaining a favourable result for a spatial unit with a truly hazardous health effect could be considered a more serious issue than vice versa.

## Conclusions

Summarising spatially structured public health data in epidemiological maps is common practice nowadays. Studying and interpreting such maps is considered a fascinating endeavour by physicians, public health researchers, policymakers, health authorities, journalists, and interested members of the general public. All parties concerned should be aware of the fact that type III errors can become a serious problem in epidemiological maps, especially for sparsely populated regions and when spatial smoothing has been applied.

## Methods

### Infant mortality in Austria

Infant mortality refers to the survival status of live births after the first year of the infant's life. In the present report infant mortality is based on individual birth certificates, which are linked with mortality records. This information is routinely collected by the statistical office of Austria [8] and is provided in an anonymized form for scientific research, i.e. no formal vote of an ethics committee is required. The statistical office of Austria [8] is an independent non-profit federal institution under public law, responsible for data collection and scientific support within the scope of federal statistics.

To calculate the infant mortality rate for a given calendar year, the number of live births that die in the first year of their lives in the respective calendar year is divided by number of live births in that calendar year. In Austria infant mortality dropped sharply from about 25.9 ‰ in 1970 to 11.2 ‰ in 1985 and 3.9 ‰ in 2010 [9,10]. Apart from the temporal trend, a clear non-random spatial distribution was observed from 1984 to 2002 [11]. In a regression model based on individual data, infant mortality in the south-eastern province of Styria was markedly lower than that in the rest of Austria even after adjusting for strong predictors like gestational age and sex. A further study based on a shared component model and stratification of mortality by cause of death confirmed these results [12].

Figure 1 illustrates the non-random spatial distribution by showing globally shrunk sex-adjusted infant mortality SMRs grouped into quartiles for the years 1984–2008 in the 121 administrative districts of Austria. Technically speaking these are empirical Bayes estimates [13] which have been computed using the Rapid Inquiry Facility (RIF, version 3.1 [14]) and depicted with ArcMap [15]. Figure 1 also shows kernel estimates of the distributions of the SMRs and expected cases of the 121 Austrian districts. The label "true SMR" is derived from the use of these values as known "true" values in the simulation study.

### Epidemiological models

It is assumed that the study region of interest (here Austria) can be partitioned into k spatial units (here k=121 districts). The true but unknown SMR for the $i^{\text{th}}$ spatial unit is denoted by $\theta_{i}$, and the observed and expected cases are denoted by $O_{i}$ and $E_{i}$, respectively. The expected cases $E_{1}\ldots E_{k}$ are considered constants, and are computed from reference mortality rates for sociodemographic strata (here overall Austrian male and female infant mortality rates from 1984 to 2008) multiplied by the corresponding strata-specific population sizes in the respective spatial units (here district-wise numbers of male and female live births for 1984–2008).

A spatial modelling approach for the observed cases is commonly based on a Poisson model [6]:

(1) $$O_{i}\sim Poisson\left(E_{i}\theta_{i}\right)\text{,}\;i=1\ldots k\text{.}$$

Depending on the parameterisation of $\theta_{1}\ldots \theta_{k}$ and the statistical approach in use (Frequentist, Bayesian), the crude, BYM and unstructured approach can be distinguished among others. Note that the correct underlying distribution is, strictly speaking, a binomial distribution for which the Poisson distribution provides a reasonable approximation as long as the event of interest is rare [6].

#### Crude SMR

In the crude case no spatial relationship is assumed, and the SMRs of spatial units are independently modelled. According to the classical Frequentist concept, SMRs are regarded as unknown population parameters that can be simply estimated by dividing observed cases by expected ones. Common Frequentist tools for statistical inference, such as p-values und confidence intervals can be applied.

Specifically, the null hypothesis $\theta_{i}=1$ is tested as follows: the actually observed number of cases in the $i^{\text{th}}$ spatial unit is used to compute an exact Poisson (1−α) confidence interval for the mean event rate $E_{i}\theta_{i}$[16,17]. Dividing the resulting lower and upper confidence limit by $E_{i}$ yields a (1−α) confidence interval for $\theta_{i}$. Note that $E_{i}$ is considered as a constant. The null hypothesis $\theta_{i}=1$ will be rejected now at the significance level α if the (1−α) confidence interval for $\theta_{i}$ does not cover the null hypothesis value of one.

#### SMR based on the BYM model

The BYM model is a seminal Bayesian model for spatial maps in health care [1,6]. The SMRs $\theta_{1}\ldots \theta_{k}$ are considered random variables with specific probability distributions. A spatial relationship can now be specified [6]:

(2) $$\log\left(\theta_{i}\right)=\delta_{i}+\nu_{i}\text{,}\;i=1\ldots k\text{.}$$

The BYM model is composed of the spatially unstructured random effect $\delta_{i}$ and the spatially structured random effect $\nu_{i}$. The unstructured component $\delta_{i}$ shrinks the estimated SMRs to the global mean, independent of the spatial configuration. By contrast, the neighbours of spatial unit i influence mean and variance of the structured component $\nu_{i}$. Therefore $\nu_{i}$ accounts for spatial dependency and shrinks the estimated SMRs to the local mean. In other words, spatial smoothing of the BYM model is due to global as well as local shrinkage.

The BYM model is estimated with the R-package INLA (version 0.1) [5,18,19]. INLA stands for Integrated Nested Laplace Approximation, and permits time-efficient Bayesian inference in latent Gaussian models with non-Gaussian response variables [18]. Default INLA values for prior distributions of model hyperparameters are used, which leads to rather non-informative and flat prior distributions. The outcome of this Bayesian approach is a bunch of posterior distributions, i.e. one for each SMR of the spatial units involved.

#### SMR based on the unstructured model

Spatial smoothing of the unstructured model is due to global shrinkage alone. Based on the specification of the BYM model above, the unstructured model may be defined as follows:

(3) $$\log\left(\theta_{i}\right)=\delta_{i}\text{,}\;i=1\ldots k\text{.}$$

The unstructured model is estimated using the R-package INLA (version 0.1) with default values for prior distribution of model hyperparameters as well.

### The type III error

Let Δ denote a true but unknown population parameter. It could be a mean, proportion, rate, difference of two means, odds ratio, SMR, or the like.

#### Classical approach

A statistical test can be used to test the null hypothesis $H_{0}:\Delta =\Delta_{0}$ at a prespecified significance level α. The corresponding non-directional two-sided alternative hypothesis is denoted by $H_{\text{A}}:\Delta \neq \Delta_{0}$.

It is difficult to imagine a practical research question for the sole existence, but not the direction of an effect. It stands to reason that the one-sided directional alternatives $H_{\text{A}1}:\Delta >\Delta_{0}$ and $H_{\text{A}2}:\Delta <\Delta_{0}$ are clearly preferred to the non-directional alternative $H_{\text{A}}$. Accordingly, the null hypothesis $H_{0}:\Delta =\Delta_{0}$ has to be replaced by the one-sided null hypotheses $H_{01}:\Delta \leq \Delta_{0}$ and $H_{02}:\Delta \geq \Delta_{0}$, respectively [3,20,21].

Carrying out a two-sided test requires the computation of a realisation t of a test statistic T, and its comparison with the lower and upper critical values $c_{\mathrm{low}}$ and $c_{\mathrm{upp}}$, respectively. These critical values depend on the selected significance level α. If $t\notin \left[c_{\mathrm{low}},c_{\mathrm{upp}}\right]$, then $H_{0}$ will be rejected for the non-directional hypothesis testing approach; if $t>c_{\mathrm{upp}}$ or $t<c_{\mathrm{low}}$, then $H_{01}$ or $H_{02}$ will be rejected for the directional approach, respectively. Note that, alternatively, a $\left(1-\alpha \right)$ confidence interval for Δ can be computed with lower and upper confidence limits $\ell_{\mathrm{low}}$ and $\ell_{\mathrm{upp}}$, respectively. Now if $\Delta_{0}\notin [\ell_{\mathrm{low}},\ell_{\mathrm{upp}}]$, then $H_{0}$ will be rejected for the non-directional hypothesis testing approach; if $\ell_{\mathrm{low}}>\Delta_{0}$ or $\ell_{\mathrm{upp}}<\Delta_{0}$ then $H_{01}$ or $H_{02}$ will be rejected for the directional approach.

Statistical power definitions for a non-directional and a directional test approach differ in respect of a minor but crucial detail [3]. If the population value $\Delta =\Delta_{1}$ is considered, without loss of generality $\Delta_{1}>\Delta_{0}$, then the type II error for the non-directional approach will be defined as $\beta_{u}={\mathrm{Pr}}_{\Delta_{1}}(c_{\mathrm{low}}\leq T\leq c_{\mathrm{upp}})$ and the non-directional power is $M_{u}={\mathrm{Pr}}_{\Delta_{1}}(T\notin [c_{\mathrm{low}},c_{\mathrm{upp}}])=1-\beta_{u}$. The directional type II error equals the non-directional one, $\beta_{d}={\mathrm{Pr}}_{\Delta_{1}}(c_{\mathrm{low}}\leq T\leq c_{\mathrm{upp}})=\beta_{u}$. However, the directional power is $M_{d}={\mathrm{Pr}}_{\Delta_{1}}(T>c_{\mathrm{upp}})$. The residual part of $M_{u}$ is the so-called type III error, $\gamma_{d}={\mathrm{Pr}}_{\Delta_{1}}\left(T<c_{\mathrm{low}}\right)=1-\beta_{d}-M_{d}=M_{u}-M_{d}$.

If the population effect is $\Delta_{1}>\Delta_{0}$ and a statistically significant result is obtained, the probability for a type III error is the so-called q-value, $q={\mathrm{Pr}}_{\Delta_{1}}(T<c_{\mathrm{low}}|T\notin [c_{\mathrm{low}},c_{\mathrm{upp}}])=\frac{M_{u}-M_{d}}{M_{u}}=\frac{\gamma_{d}}{M_{u}}$, which is the type III error proportion of the non-directional power [3]. In the case of $\Delta_{1}<\Delta_{0}$ the definitions have to be appropriately adapted and $q={\mathrm{Pr}}_{\Delta_{1}}(T>c_{\mathrm{upp}}|T\notin [c_{\mathrm{low}},c_{\mathrm{upp}}])$. The *q*-value should be small, e.g. q≤0.01, or better still q≤0.001[3].

#### Bayesian approach

The type III error has been considered so far in the framework of the classical (Frequentist) approach to statistics. A Bayesian approach would yield a posterior distribution f(Δ|data) for the unknown parameter Δ. The posterior distribution may be used for decision-making by computing $\mathrm{Prob}(\Delta >\Delta_{01})$ from it [6]. Here $\Delta_{01}$ is known as the reference threshold and is not necessarily equal to the null hypothesis value $\Delta_{0}$ from above. Now choose a cut-off probability $\omega_{\;1}$, e.g. $\omega_{\;1}=0.8$ or $\omega_{\;1}=0.975$, and classify Δ as "significantly" increased if $\mathrm{Prob}(\Delta >\Delta_{01})>\omega_{\;1}$. A two-sided decision approach requires the corresponding definition of a "significant" decrease, that is, $\mathrm{Prob}(\Delta <\Delta_{02})>\omega_{2}$. Note that $\mathrm{Prob}(\Delta >\Delta_{01})>\omega_{\;1}$ and $\mathrm{Prob}(\Delta <\Delta_{02})>\omega_{2}$ are known as decision rules.

Computation of the directional and non-directional power, the type III error and the *q*-value is now straightforward. Here the meaning is obvious from the context. However, the term "significant" is not common in Bayesian statistics. Therefore, for the sake of clarity "significant" is mentioned in quotation marks in connection with a Bayesian result throughout the present report.

### Simulation study

Directional power, type III error and the *q*-value of the two random effect models were numerically approximated by computer simulations using the R-package INLA (version 0.1) [5,18,19]. Austrian infant mortality data from 1984 to 2008 were used for exemplification. The empirical Bayes estimates [13] of the SMR values shown in Figure 1 were assumed to be the known true population values $\theta_{i}$, i=1…121 ("true SMRs"). It should be noted that none of the $\theta_{i}$'s equals one.

Expected cases $E_{i}$ for each of the 121 districts were calculated as the sum of expected male and female cases. The expected male cases were obtained by multiplying the number of male live births in the $i^{\text{th}}$ district for the years 1984–2008 by the overall Austrian male infant mortality rate for 1984–2008. The expected female cases were obtained by analogy.

Based on the expected cases $E_{i}$ and the "true SMR" values $\theta_{i}$, realisations of the observed cases $O_{i}$, i=1…121, were derived from a multinomial distribution, as described by Richardson et al. in appendix A of their report [6]. This procedure ensured that, for each of the 100,000 iterations of the simulation study, the sum of the generated observed cases remained at the same value of 13,294, which is the total number of deceased infants in Austria from 1984 to 2008. Richardson et al. [6] note that this procedure changes the "true SMRs" by a constant which is 1.00026 in this instance. The issue was ignored in the following as the effect is very small and, in particular, the situation $\theta_{i}<1$ but $1.00026\times \theta_{i}\geq 1$ never occurred for i=1…121.

The expected cases and the simulated realisations of the observed cases were used to estimate the unstructured and the structured random effect model. Each of these Bayesian models yielded posterior distributions for the 121 district-wise SMR values. The type III error for a district was obtained by determining how often a “significant” result on the opposite side of the districts' true SMR value was observed for each of the 100,000 iterations of the simulation study. Dividing the type III error by the number of overall “significant” results (i.e. the non-directional power) for the district yielded the districts' *q*-value.

## Competing interests

Both authors declare that they have no competing interests.

## Authors’ contributions

Both authors contributed equally to this research, and read and approved the final manuscript.
